# A novel YAP1/SLC35B4 regulatory axis contributes to proliferation and progression of gastric carcinoma

**DOI:** 10.1038/s41419-019-1674-2

**Published:** 2019-06-07

**Authors:** Jun Liu, Xinhui Zhao, Ke Wang, Xiang Zhang, Yanping Yu, Yongzhi Lv, Shun Zhang, Long Zhang, Yuan Guo, Yunlong Li, Angang Yang, Rui Zhang, Jipeng Li

**Affiliations:** 1 State Key Laboratory of Cancer Biology, Institute of Digestive Diseases, Xijing Hospital, Fourth Military Medical University, 710032 Xi’an, China; 2 State Key Laboratory of Cancer Biology, Department of Biochemistry and Molecular Biology, Fourth Military Medical University, 710032 Xi’an, China; 3 School of Clinical Medicine, Xi’an Medical University, 710032 Xi’an, China; 4 State Key Laboratory of Cancer Biology, Department of Immunology, Fourth Military Medical University, 710032 Xi’an, China

**Keywords:** Gastric cancer, Tumour biomarkers

## Abstract

Solute carrier family 35 member B4 (SLC35B4), a nucleotide sugar transporter, is capable of transporting UDP-xylose and UDP-GlcNAc from the cytoplasm to the lumen of the endoplasmic reticulum and Golgi. SLC35B4 has a pivotal role in glycosylation of biological macromolecules. However, its functional roles and regulatory mechanisms in malignant diseases remain unknown. Here, using the cDNA arrays, promoter reporter assays, and chromatin immunoprecipitation assays, we demonstrated that SLC35B4 is directly transactivated by YAP1–TEADs complex in gastric cancer (GC) cells. CCK-8, plate colony formation and soft agar assays revealed that SLC35B4 is essential for survival and proliferation in GC cells and nude mice models. SLC35B4 expression is markedly higher in GC tissues compared with control noncancerous tissues. Immunohistochemistry revealed that SLC35B4 expression is positively correlated with YAP1 expression in human GC tissues, and this correlation is also confirmed in the GC TCGA data set. GC patients with high levels of SLC35B4 expression have poorer prognosis than those with low levels of SLC35B4 expression. Collectively, our findings defined SLC35B4 as an important downstream oncogenic target of YAP1, suggesting that dysregulated signaling of a novel YAP1/SLC35B4 axis promotes GC development and progression, and this axis could be a potential candidate for prognosis and therapeutics in GC.

## Introduction

Gastric cancer (GC) is a leading cause of global cancer mortality, especially in the Eastern Asian countries^1^. Although the current therapeutics, including surgical resection and chemotherapy, have improved the overall prognosis of clinical patients with GC, a high rate of recurrence and a substantially low survival rate in advanced cancer have not been solved definitely^2^. Molecular and genetic alterations in GC have provided critical information regarding optimal timing and treatment regimens^3,4^. However, to date, analysis of its molecular and clinical characteristics has been complicated only by histological and etiological heterogeneity. It cannot be satisfied with the requirement of precision medicine for the GC treatment. Recently, according to the molecular signatures, GC has been divided into four subtypes: Epstein–Barr virus-positive, microsatellite instability, genomic stability, and chromosomal instability^5,6^. Thus, there is an urgent need to deeply explore the underlying molecular mechanisms for initiation and progression of GC, to offer more clues for novel therapeutics and to identify better biomarkers for prognosis.

The Hippo-YAP signaling pathway has been demonstrated to have a critical role that controls tissue homeostasis and organ size^7^. YAP1 (Yes-associated protein 1) is a major downstream effector of the Hippo pathway^8^. In the high activation of this pathway, the complex of macrophage stimulating 1/2 (MST1/2) phosphorylates Last1/2 kinases, and consequently results in phosphorylation of YAP1 on five S/T amino-acid residues with a consensus sequence. The activity of YAP1 is blocked by 14-3-3 protein-mediated retention in the cytoplasm or E3 ligase-mediated protein degradation. When Hippo signaling pathway is inhibited, the MST1/2-Last1/2 kinase cascade is blocked^9–11^. The non-phosphorylated YAP1 translocates into nucleus and physically interacts with the DNA-binding protein TEA domain transcription factors (TEADs), and then transcriptionally activates downstream genes such as CTGF, Cyr61, and etc.^12–14^.

In recent years, a growing body of evidence shows that the Hippo-YAP signaling pathway is closely associated with development and progression of solid tumors. YAP1 is a core component of the Hippo signaling pathway implicated in tumorigenesis, and activated YAP1 functions as a driver oncogene in multiple cancer types^15,16^. Transforming properties of YAP1 were first reported in mammary epithelial cells. Overexpression of YAP1 can induce epithelial–mesenchymal transition (EMT) and increase colony capability in MCF-10A cells in vitro^17^. And a serial of studies have demonstrated that a high YAP1 activation dysregulates multiple oncogenic signaling pathways. In hepatocellular carcinoma (HCC), YAP1 cooperates with forkhead box M1 to drive chromosomal instability-related gene expression^18^. Meanwhile, YAP silencing in HCC restores hepatocyte differentiation and leads to tumor regression^19^. Increased YAP1 promotes resistance to RAF (Raf-1 proto-oncogene) or mitogen-activated protein kinase kinases inhibitor therapy through transcription active of an anti-apoptotic protein BCL2 like 1 in many types of tumor cells^20^. YAP-dependent Jag-1 triggers activation of Notch signaling, and that sequential activation of YAP and Notch signaling mediates YAP-dependent effects on tumor progression in HCC, colorectal and pancreatic cancers^21^. In recent years, more evidence reveals the critical roles of YAP1-mediated cross-talking with Notch or β-catenin signaling pathways are responsible for chronic inflammation and carcinogenesis^22,23^.

In general, YAP1 functions as a transcription co-activator and TEAD transcription factors are the main binding partner for YAP1, together they exert oncogenic roles in tumorigenesis^24^. However, the downstream players of YAP1/TEAD complex in GC are unidentified systematically. To date, the clinicopathologic significance of SLC35B4 in GC has not been reported. In the present study, we provided the first evidence that SLC35B4, which is transactivated by YAP1–TEADs complex, promotes GC cells survival and proliferation, and is closely associated with poor prognosis of GC patients. SLC35B4 expression is positively correlated with YAP1 expression in our collected human GC tissues and TCGA-derived data set. Collectively, our study demonstrated that a novel YAP1/SLC35B4 axis may have an important role in tumorigenesis of GC, and it could be a novel prognostic biomarker and a potential candidate for future therapeutic applications in GC.

## Results

### YAP1 promotes proliferation of GC cells in vitro and in vivo

**Fig. 1:**
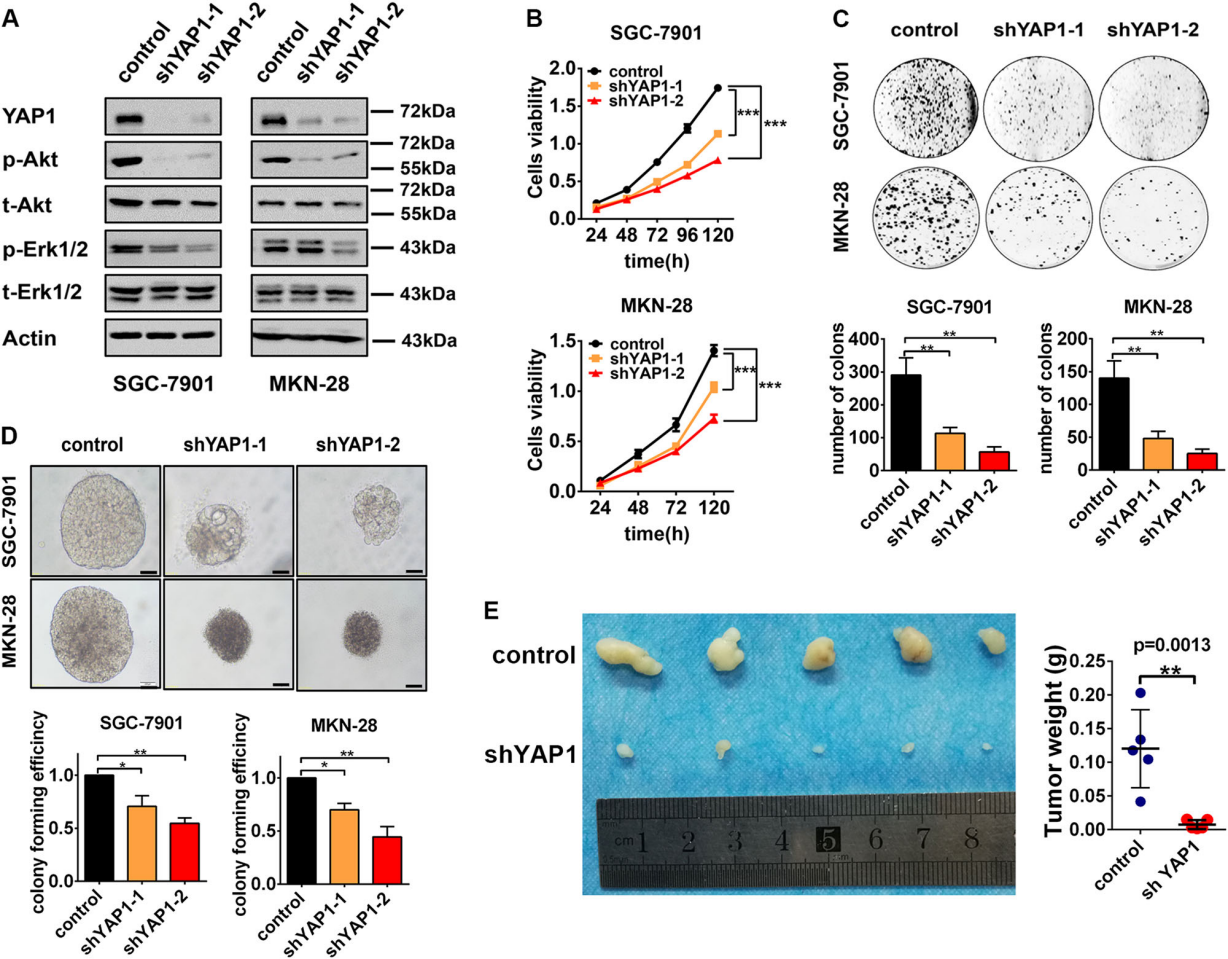
YAP1 promotes proliferation of gastric cancer cells in vitro and in vivo. **a** SGC-7901 and MKN-28 cells were infected with lentvirus expressing shRNA specific to YAP1 or scramble shRNA. The protein levels of p-Akt, t-Akt, p-Erk1/2, t-Erk1/2, and Actin were detected by western blot. The western blot analysis was performed two times. **b** Cell viability of SGC-7901 and MKN-28 cells was detected by Cell Counting Kit-8 assay. **c** Plate colony formation assay and **d** soft agar colony formation assay of GC cells (scale bar is 100 µm). Cell Cycle Kit-8 assay, plate colony formation assay and soft agar colony formation assay were performed three times. **e** Tumor volume (left) and tumor weight (right) of SGC-7901 cells from nude mice with subcutaneous infection. Control group, *n* = 5, shYAP1 group, *n* = 5

YAP1 is reported as an oncogene in most of malignant solid tumors including GC. To confer the function of YAP1 in GC cells, we generated YAP1-knockdown SGC-7901 and MKN-28 cell lines using two YAP1-specific shRNAs by lentivirus infection and control cells infected by the lentivirus containing a scramble shRNA. Western blotting assays showed that the YAP1 expression is efficiently knocked down in both SGC-7901 and MKN-28 GC cell lines (Fig. 1a). To investigate the influence of YAP1 on survival and proliferation in GC cells, we performed CCK-8 assay, plate colony formation assay, and soft agar colony formation assay. The viability of cells reduces in YAP1-knockdown cells in CCK-8 assay (Fig. 1b). Moreover, the colony formation ability is also attenuated in YAP1-knockdown cells (Fig. 1c). And a similar result is gained in soft agar colony formation experiments, shown as the decreases of sphere size and colony forming efficiency in the YAP1-knockdown cells, compared with control cells (Fig. 1d). To confer the function of YAP1 in vivo, we subcutaneously injected SGC-7901 in nude mice. The growth of tumor is substantially suppressed by YAP1-knockdown, shown as the tumor volume and tumor weight are reduced in YAP1-knockdown group (Fig. 1e). Considering that the phosphorylated Akt and Erk1/2 has an essential role for cell proliferation^25,26^, we analyzed the phosphorylation levels of Akt and Erk1/2. Western blot analysis showed that the levels of phosphorylated Akt and Erk1/2 are decreased in YAP1-knockdown cells compared with the control cells (Fig. 1a), whereas there is no obvious difference on the expression levels of total Akt and Erk1/2 between control cells and YAP1-knockdown cells (Fig. 1a). These results demonstrated that YAP1 could function as a driver to promote survival and proliferation in GC cells by upregulating Akt and Erk1/2 kinases activation, further confirming the important role of YAP1 in GC cells.

### Screening and identification of SLC35B4 as a novel downstream gene regulated by YAP1 in GC cells

**Fig. 2:**
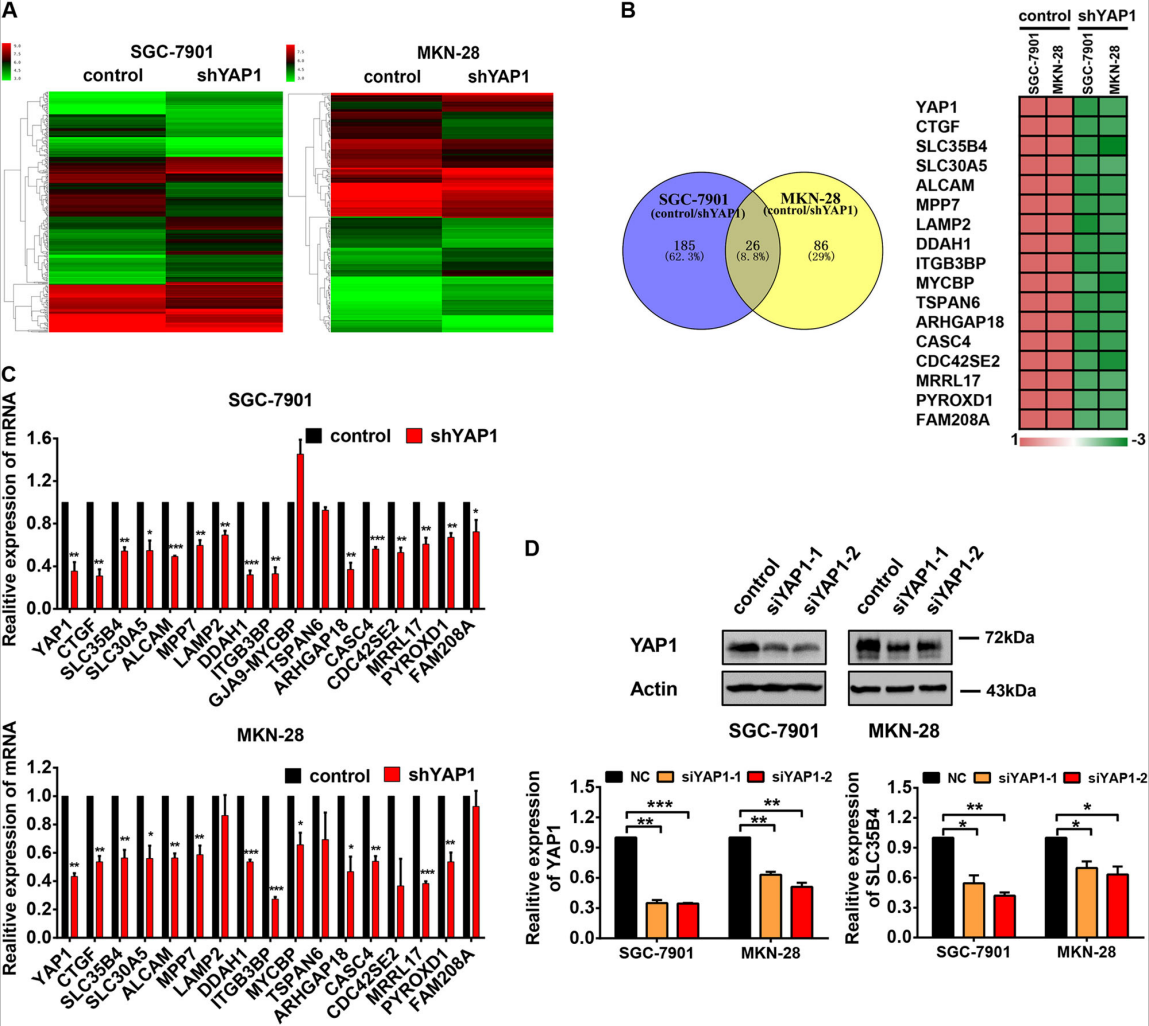
Screening and identification of SLC35B4 as a novel downstream gene regulated by YAP1 in gastric cancer cells. **a** Heat map of differentially expressed genes between control (infection with lentvirus expressing scramble shRNA) and YAP1-knockdown (infection with lentvirus expressing shYAP1-2) cells. **b** Comparative intersection analysis of downregulated genes in YAP1*-*knockdown SGC-7901 and MKN-28 cells. **c** Seventeen candidated genes were validated in YAP1-knockdown SGC-7901 and MKN-28 cells by qRT-PCR. This experiment was performed three times. **d** Protein level of YAP1 and mRNA level of SLC35B4 in SGC-7901 and MKN-28 cells with transfection of YAP1 siRNAs. This experiment was performed three times

To investigate the underlying molecular mechanism of YAP1-mediated regulation in GC cell proliferation, microarray assay was used in SGC-7901 and MKN-28 GC cell lines to find possible downstream genes regulated by YAP1. The heat map of gene expression profiles showed that mRNA levels of various genes are obviously changed in YAP1-knockdown cells compared with that in control cells (Fig. 2a). As YAP1 has been known as a transcriptional co-activator, we focused on downregulated genes in YAP1-konckdown cells, and an overlap in the Venn diagram showed that 26 probes representing 17 genes are downregulated in YAP1-knockdown GC cell lines (Fig. 2b). *CTGF*, the well-documented target gene of YAP1, has been found in our microarray data, further confirming the reliability for our experiments (Fig. 2b). And then, we performed the RT-qPCR to validate our finding from microarray assays (Fig. 2c). Among all validated YAP1-activated genes in our experiments, SLC35B4 encodes a bifunctional nucleotide sugar transporter (NST) with specificity for UDP-Xylose and UDP-*N*-acetylglucosamine. To verify the regulation of SLC35B4, we determined the mRNA level of SLC35B4 in GC cells transfected with YAP1 siRNAs. The expression of SLC35B4 decreases in GC cells transfected with YAP1 siRNAs (Fig. 2d). However, its expression pattern, biological functions, and regulatory mechanisms in transformed cells are unknown.

### Inhibition of SLC35B4 suppresses proliferation of GC cells in vitro and in vivo

**Fig. 3:**
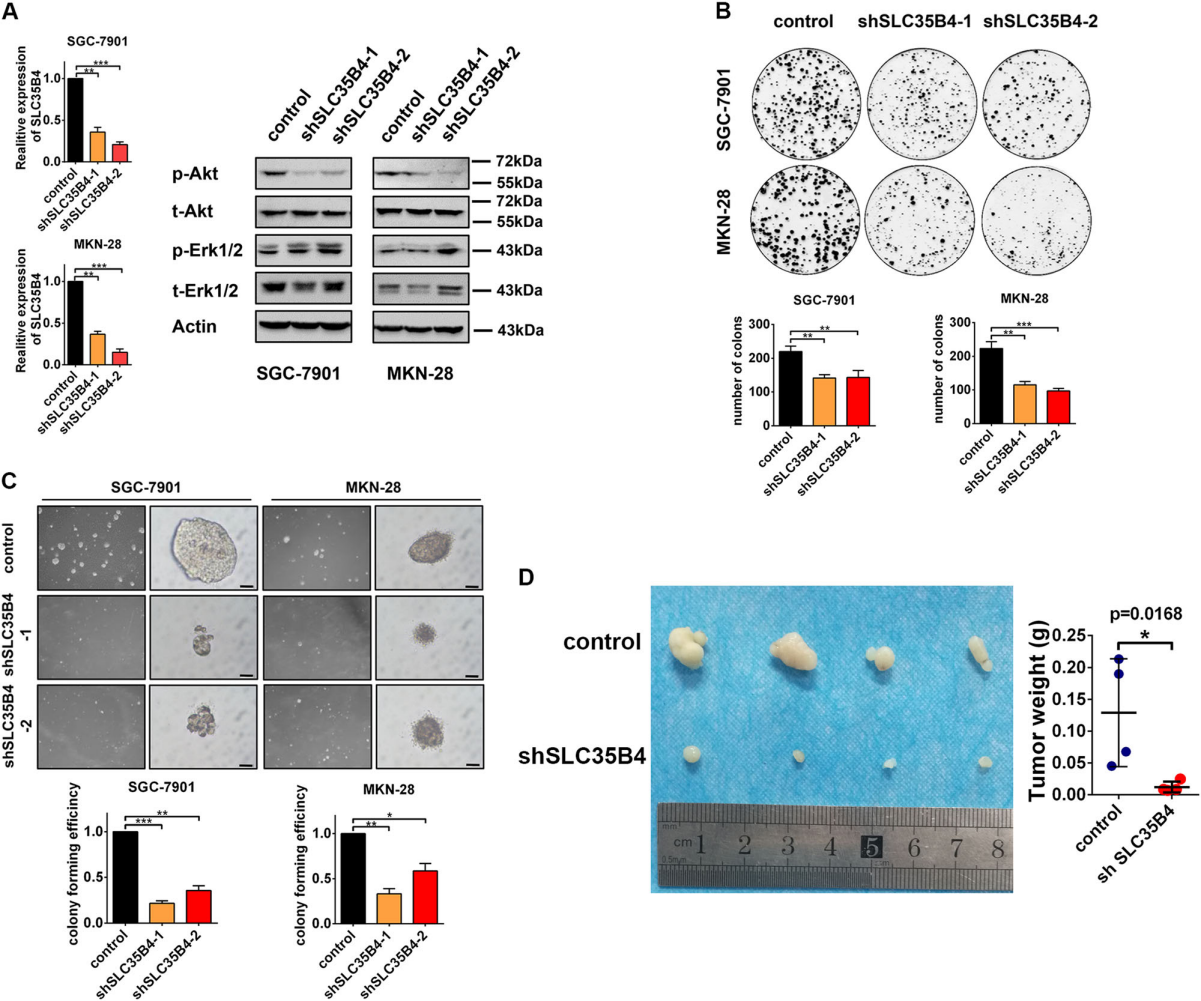
Inhibition of SLC35B4 suppresses proliferation of gastric cancer cells in vitro and in vivo. **a** SGC-7901 and MKN-28 cells were infected with lentvirus expressing SLC35B4-shRNA or scramble shRNA. Protein levels of p-Akt, t-Akt, p-Erk1/2, t-Erk1/2, and Actin were monitored by western blot. mRNA level of SLC35B4 was detected by RT-qPCR. The experiment was repeated three times. **b** Colony formation assay and **c** soft agar colony formation assay of SGC-7901 and MKN-28 cells was performed to determine the cell viability. The scale bar is 100 µm. These experiments were performed three times. **d** Tumor volume (left) and tumor weight (right) of SGC-7901 cells from nude mice with subcutaneous infection. Control group, *n* = 4, shSLC35B4 group, *n* = 4

According to our finding that high level of SLC35B4 could be an independent biomarker for poor prognosis of GC patients and it is transcriptionally regulated by YAP1–TEADs complex, we wondered to know the functional role of SLC35B4 in GC cells. We generated SLC35B4-knockdown SGC-7901 and MKN-28 cell lines by a lentiviral system expressing SLC35B4-targeting shRNAs. As there is no suitable commercial antibody for western blot, we had to perform RT-qPCR to determine the expression of SLC35B4 in two GC cell lines (Fig. 3a). Western blot analysis showed that the expression level of phosphorylated Akt decreases significantly in SLC35B4-knockdown cells (Fig. 3a). The results from plate colony formation assay and soft agar colony formation assay (Fig. 3b, c) revealed that SLC35B4 contributes to cell proliferation in GC cells, as both number of clones and size of spheres reduced when SLC3B4 was deficient. In the in vivo experiment, we subcutaneously injected SGC-7901 cells in nude mice. The volume and weight of xenografts were measured. The result showed that knockdown of SLC35B4 inhibits tumor growth compared with the control group (Fig. 3d). All these results suggested that SLC35B4 might function as an oncogenic molecule in GC cells in vitro and in vivo.

### YAP1 promotes proliferation of GC cells dependent on SLC35B4 partially

**Fig. 4:**
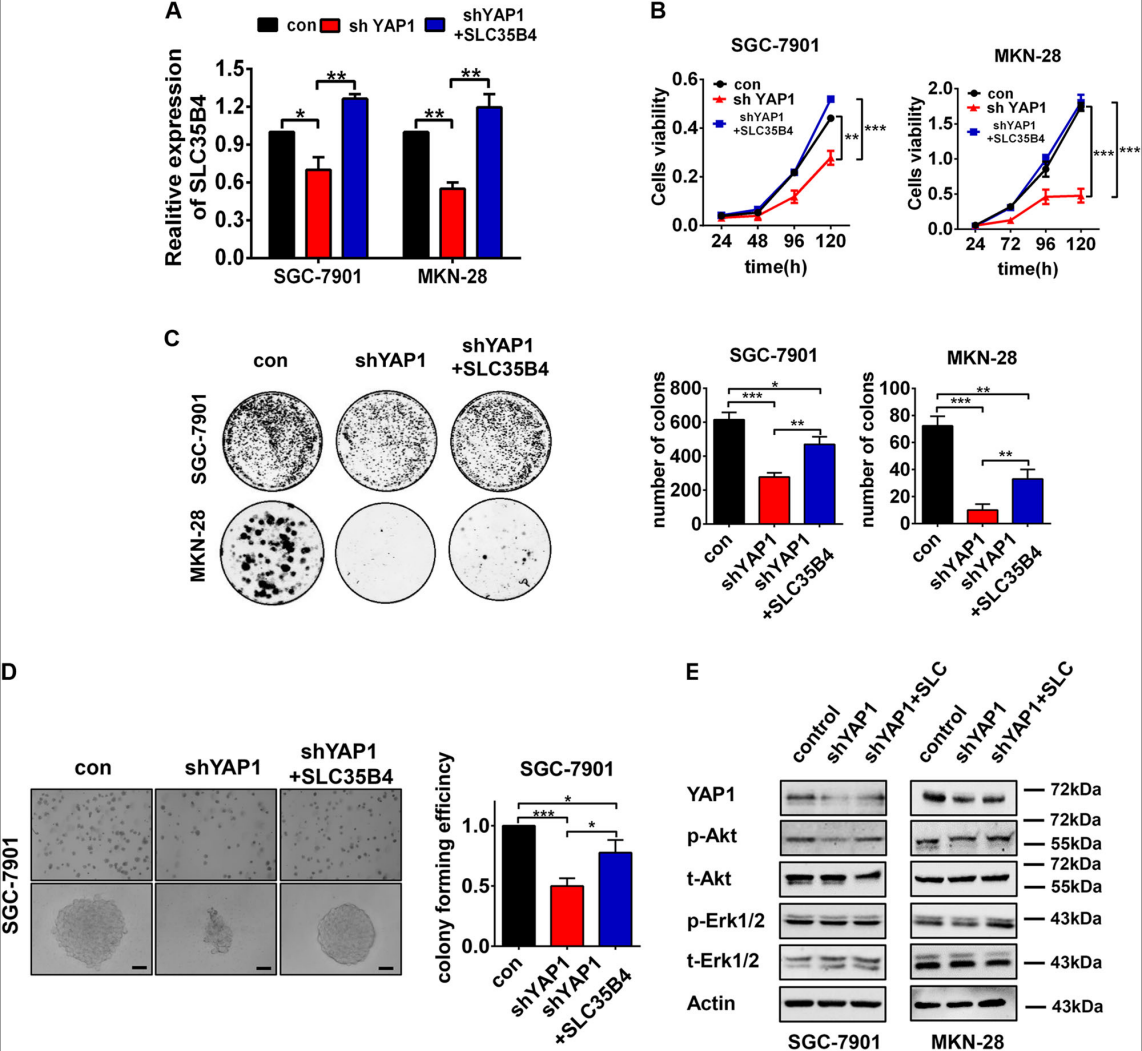
YAP1 promotes the proliferation of gastric cancer cells dependent on SLC35B4 partially. **a** RT-qPCR was performed to detect SLC35B4 expression in SGC-7901 and MKN-28 cells infected with lentvirus expressing YAP1-shRNA and full-length of SLC35B4 gene. This experiment was performed three times. **b** CCK-8 assay of SGC-7901 and MKN-28 cells infected with lentvirus expressing scramble shRNA (control), shYAP1 and shYAP1 combined with SLC35B4 overexpression. This experiment was performed three times. **c** Colony formation assay of GGC-7901 and MKN-28 cells and **d** soft agar colony formation assay of SGC-7901 cells were performed to determine the cell viability. The scale bar is 100 µm. These experiments were performed three times. **e** Protein levels of YAP1, p-Akt, t-Akt, p-Erk1/2, t-Erk1/2, and Actin were monitored by western blot. This experiment was performed two times

To determine the biological significance of SLC35B4 in YAP1-mediated GC cell proliferation, we performed the rescue experiment by reintroduction of SLC35B4 in GC cells expressing a YAP1-shRNA. As shown in Fig. 4a, RT-qPCR analysis showed obvious upregulation of the exogenous SLC35B4 expression in YAP1-silencing GC cells. In the functional experiments, analysis of cell counting and colony formation assays showed that GC cells grow faster in the SLC35B4 overexpressing group compared with it in the control group (Fig. 4b, c). Moreover, the soft agar colony formation assay in SGC-7901 cells showed that overexpression of SLC35B4 stimulates cell proliferation in YAP1-knockdown cells (Fig. 4d). Finally, western blot analysis showed that protein level of phosphorylated Akt is recovered in SLC35B4 reintroduced cells compared with it in the YAP1-knockdown cells (Fig. 4e). Our results demonstrated that a novel YAP1-SLC35B4 axis contributes to proliferation in GC cells.

### The expression of SLC35B4 is transcriptionally activated by the YAP1-TEADs complex in GC cells

**Fig. 5:**
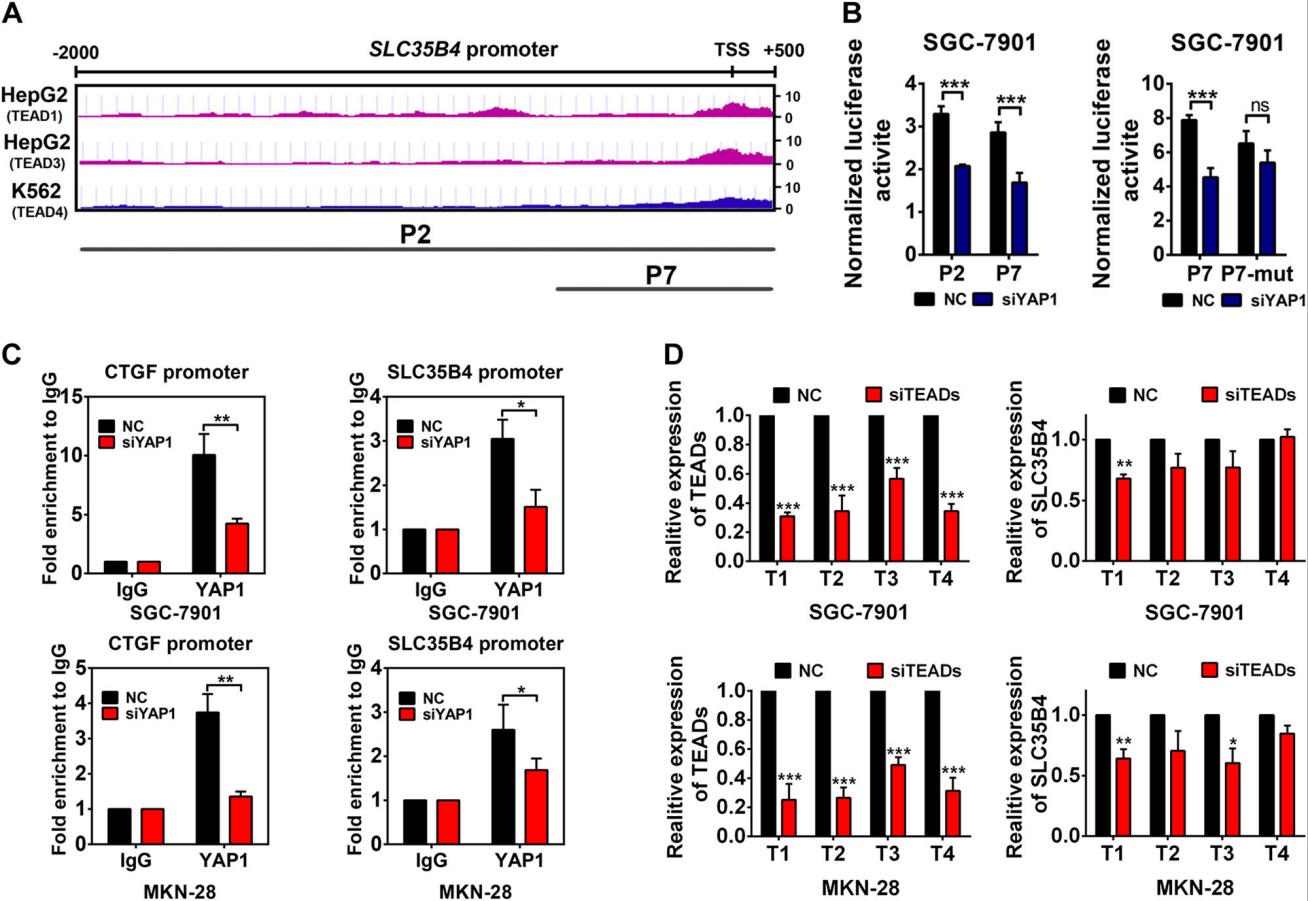
The expression of SLC35B4 is transcriptionally activated by the YAP1-TEADs complex in gastric cancer cells. **a** The TEADs-binding signal in SLC35B4 promoter were analysis using ENCODE ChIP-Seq database. **b** SGC-7901 cells were transfected with YAP1 siRNAs and pGL3 plasmid inserted with sequence of SLC35B4 promoter before Dual-Luciferase report analysis was performed. P2, full-length promoters of SLC35B4; P7, the truncated promoter of SLC35B4; P7-mut, the mutation of SLC35B4 promoter. These experiments were performed three times. **c** ChIP-qPCR analysis in SGC-7901 and MKN-28 cells. The enrichment of CTGF promoter was performed as a positive control. This experiment was performed three times. **d** RT-qPCR analysis of TEADs and SLC35B4 in SCG-7901 and MKN-28 cells transfected with siTEAD1/2/3/4 separately. T1, TEAD1; T2, TEAD2; T3, TEAD3; T4, TEAD4. The experiment was performed three to four times

YAP1 has been known as a transcriptional co-activator to regulate gene expression involved in cell proliferation and tissue growth combined with DNA-binding protein TEADs (TEAD1–4). We supposed that SLC35B4 might be transcriptionally activated by YAP1–TEADs in GC cells. Considering the YAP1–TEADs complex is capable of binding with DNA directly, we searched for potential promoter sequences that contain the TEADs-binding sites in ENCODE chromatin immunoprecipitation (ChIP)-Seq database. We found one potential TEADs-binding site on the promoter region of SLC35B4 as shown in Fig. 5a. To verify a proper transcriptional mechanism of YAP1 in the regulation of SLC35B4 expression, we cloned full-length and truncated forms of SLC35B4 promoter regions. A promoter report assay showed that the luciferase activity driven by both the full-length and the truncated promoters of SLC35B4 decrease in SGC-7901 cells transiently transfected with YAP1-specific siRNAs (Fig. 5b). We mutated the potential TEADs-binding sites in the truncated promoter of SLC35B4, the activity of mutated promoter showed no significant changes in YAP-knockdown cells compared with that in the control group (Fig. 5b). It indicated that YAP1–TEADs can bind with SLC35B4 promoter and promote the transcriptional activity of SLC35B4 promoter. To further confirm whether YAP1 can bind with SLC35B4 promoter directly in vivo, we performed a ChIP assay in GC cells. qPCR of TEADs-binding chromatin showed a recruitment of YAP1 on the promoter region of SLC35B4 (Fig. 5c). Meanwhile, siRNAs targeting TEADs were transfected into GC cells to figure out whether TEADs could regulate expression of SLC35B4 in GC cells. Our data in Fig. 5d showed that only TEAD1 silencing has a significantly inhibitory effect on the expression of SLC35B4 in two tested cell lines, whereas knockdown of TEAD3 can also inhibit the SLC35B4 expression in only MKN-28 cells. These phenomena may be because of the different cellular context of GC cells and the diverse abundance of TEADs in GC cell lines. Collectively, our results demonstrated that SLC35B4 is a novel downstream gene transcriptionally regulated by YAP1–TEADs complex in GC cells.

### The relationship between SLC35B4 and YAP1 in GC

**Fig. 6:**
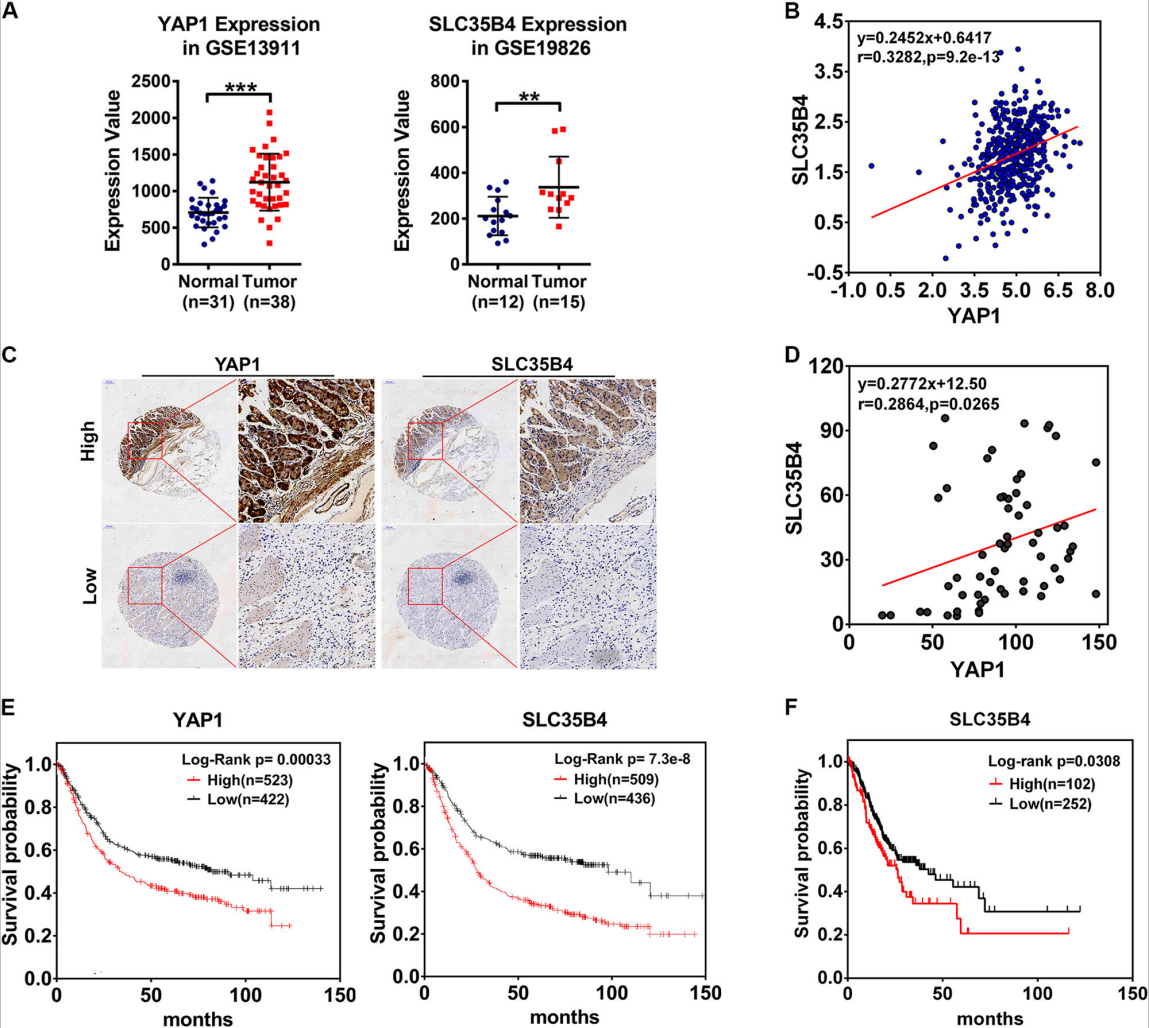
The relationship between SLC35B4 and YAP1 in gastric cancer. **a** Analysis of YAP1 expression in normal tissue and gastric tumor from Derrico Gastric data set (GSE13911, N, normal gastric mucosa, *n* = 31; T, gastric tumor, *n* = 38) and SLC35B4 expression from Wang Gastric data set (GSE19826, N, normal gastric mucosa, *n* = 15; T, gastric tumor, *n* = 12). **b** The mRNA expression patterns of YAP1 and SLC35B4 in 450 gastric cancer samples. **c** Protein levels of YAP1 and SLC35B4 in human GC tumor tissues were monitored by IHC analysis. **d** The tissue array of 60 human GC tissue samples was analyzed by IHC. The scale bar is 200 µm. The correlation analysis of YAP1 and SLC35B4 was decided by histochemistry score of 60 human GC tissue samples. **e** The correlation between YAP1 expression and overall survival of gastric cancer patients was analyzed by Kaplan–Meier Plotter. **f** Survival analysis of TCGA GC patients. The survival days and mRNA expression data of SLC35B4 of 354 gastric cancer patients was downloaded from TCGA. (high expression of SLC35B4 group, *n* = 102; low expression of SLC35B4 group, *n* = 252. Expression of SLC35B4 cutoff value was 3.35 FPKM)

To determine the relationship of YAP1 and SLC35B4 expression levels in human GC, we analyzed the expression pattern of YAP1 and SLC35B4 in the GEO database, the RNA levels of YAP1 showed an abnormal increase in gastric tumors compared with normal tissues and SLC35B4 also had a similar expression pattern in GC tissues as shown in Fig. 6a. Furthermore, we analyzed the relationship of the mRNA levels of YAP1 and SLC35B4 in the TCGA data set of human GC. The result revealed a closely positive correlation between YAP1 and SLC35B4 RNA levels in 450 clinical samples (Fig. 6b). To further confirm the relationship between YAP1 and SLC35B4 in the protein levels, we performed an immunohistochemistry (IHC) staining to observe the expression patterns of YAP1 and SLC35B4 in a GC tissue array. As showed in Fig. 6c, the level of SLC35B4 frequently increases in YAP1-high-expressing GC tissues. Statistic analysis indicated a positive correlation between YAP1 and SLC35B4 in the 60 GC samples (Fig. 6d). Taken together, our data confirmed a co-expression pattern of YAP1 and SLC35B4 in GC tissues and further validated the existence of a YAP1/SLC35B4 regulatory axis in GC.

## Discussion

### Aberrantly high activation of YAP1 is closely associated with development and progression of GC

Hippo signaling pathway is an emerging kinase cascade in gastrointestinal homeostasis and tumorigenesis^27,28^. As the main target for Hippo pathway, the oncogenic role of YAP1 has been extensively investigated^15,16^. In recent years, a growing body of evidence has demonstrated a potential link between YAP/TAZ signaling and GC^29^. In the clinical samples, YAP expression is upregulated in gastric tumor tissues compared with normal tissues, and the high levels of YAP expression as well as its nuclear localization are significantly correlated with poor prognosis in GC patients^30,31^. Strikingly, Zhou’s group designed a peptide mimicking the role of VGLL4 (an inhibitor for the YAP-TEAD interaction) to suppress the tumor growth in both xenograft and carcinogen-induced murine gastric tumor models^32^. More importantly, a genetically engineered mouse model has been established to determine the important role of YAP/TAZ activation in development and progression of GC. Hippo activator Lats1/2 was deleted from LGR5-positive cells, and consequently activated YAP/TAZ. The phenotype analysis showed this perturbation was sufficient to trigger dysplastic changes and eventually tumorigenesis in the gastric epithelium^33^. A pathogenic protein *Helicobacter pylori* CagA can promote gastric tumorigenesis by activating oncogenic YAP and promote EMT of GC^34^. Taken together, all these evidence support that the aberrantly high activation of YAP1 is closely associated with development and progression of GC. However, the systematic significance of Hippo-YAP/TAZ signaling in GC has not been established in the transcriptomic levels. In the present study, using the loss-of-functional experiments, we silenced the YAP1 expression in GC cell lines. Based on our functional data, we confirmed an oncogenic role of YAP1 in GC cells. To investigate the underlying molecular mechanism of YAP1-mediated oncogenic functions in GC cells, we used a complementary DNA (cDNA) array to systematically screen and identify the putative downstream genes regulated by YAP1 in our established GC cell lines. Among the 17 genes that displayed decreased expression in the YAP1-knockdown GC cells, SLC35B4 attracted our attention because there is almost no any report of this gene in malignant diseases.

### SLC35B4 is a novel downstream gene transcriptionally activated by YAP1–TEADs complex

As a co-transcription factor, YAP1 binds with DNA-binding protein TEADs to form a transcriptional complex, and consequently binds to the promoter of downstream effector genes and stimulates their transcriptional activities^24^. In the present study, by combinationally using the Jaspar Transfactor Prediction Software and ENCODE ChIP data set, we found a putative DNA-binding site of YAP1–TEADs on the promoter region of SLC35B4. By using the promoter luciferase assay and ChIP-qPCR, we first revealed that SLC35B4 is a novel downstream gene directly regulated by YAP1/TEADs in gastric carcinoma cells. In addition, the data from IHC staining on tissue microarray and RNAseq analysis from TCGA data set both identified a closely correlation between SLC35B4 and YAP1 in the protein and mRNA levels. All these results further confirmed the relationship between SLC35B4 and YAP1 in the gastric carcinoma.

Solute carrier family 35 member B4 (SLC35B4), one of NSTs, belongs to solute carrier (SLC) super family which helps for transporting various biological molecules to pass through cell or organelle membranes^35^. Functionally, UDP-xylose and UDP-GlcNAc can be transported by SLC35B4 from cytoplasm into the lumen of the endoplasmic reticulum (ER) and Golgi apparatus and then be utilized by glycosyltransferases^36^. SLC35B4 was cloned and firstly reported in 2005^35^, but in the past one decade, there was almost no any report on its biological functions except few studies demonstrated that it is involved in the regulation of obesity, insulin resistance and gluconeogenesis^37,38^. Here, we have identified SLC35B4 is a downstream gene directly regulated by YAP1 in GC cells. Our finding indicated that it may be involved in YAP1-mediated proliferation in GC cells. However, if SLC35B4 is a context-dependent target gene or a general target gene of YAP1 still needs to be further confirmed in multiple cancers in the future.

### A novel YAP1/SLC35B4 regulatory axis contributes to proliferation and progression of GC

As an oncogenic transcriptional factor, previous studies have demonstrated that YAP1 promotes cell proliferation and inhibits apoptosis in cancer cells by transcription activating of growth factor (e.g., CTGF) or anti-apoptotic proteins (e.g., Bcl2l1)^13,20^. Here, we identified that SLC35B4 is a novel downstream gene transcriptionally activated by YAP1 in GC cells. In the transformed cells, cellular metabolism involving glycosylation of proteins is usually more frequent, a higher level of NSTs can ensure the sufficient substrate supply for glycosylation of proteins, lipids, and proteoglycans^39^. A recent study pointed out that UDP-GlcNAc can act as a donor sugar of O-GlcNAc transferase, which O-GlcNAcylates YAP at Ser109, and finally prevents YAP phosphorylation by LAST1. And the O-GlcNAcylated YAP promotes cancer cell growth in vitro and in vivo by its transcriptional activity^40^. And another study shows that YAP O-GlcNAcylation at Thr241 promotes liver tumorigenesis by inhibition of β-TrcP^41^. It implies that SLC35B4 may have an important role in tumorigenesis by ensuring the sufficient donor sugar for YAP O-GlcNAcylation. According to this logical reasoning combined with our experiments in this study, it can help us to deeply understand the biological significance of a YAP1/SLC35B4 axis in the regulation of malignant behaviors in gastric carcinoma. More importantly, our study investigated the prognostic potential of YAP1/SLC35B4 axis expression in survival of patients with GC, supporting the prognostic value of YAP1 and SLC35B4 expression, which allows clinicians to potentially identify candidate patients for appropriate treatment to improve therapeutic outcomes.

Collectively, these findings demonstrate that YAP1 transcriptionally activates the expression of SLC35B4 to promote cell survival and proliferation and progression of GC. Our study confirms the important prognostic value of a YAP1/SLC35B4 axis in GC and highlights the essential role of SLC35B4 in the YAP1-driven progression of GC. Thus a novel YAP1/SLC35B4 axis sheds light upon a potential prognostic and therapeutic value for GC patients in the future.

## Materials and methods

### Cell culture

Human GC cell lines, SGC-7901 and MKN-28, were cultured in Rosewell Park Memorial Institute Medium 1640 (GIBCO BRL, Grand Island, NY, USA) supplemented with 10% fetal bovine serum (GIBCO BRL), penicillin (100 milligram per milliliter, mg/ml), and streptomycin (100 mg/ml). HEK293T cells were cultured in Dulbecco’s Modified Eagle Medium (GIBCO BRL) supplemented with 10% fetal bovine serum (GIBCO BRL), penicillin (100 mg/ml) and streptomycin (100 mg/ml). Cells were incubated at 37 °C with 5% CO_2_. Human GC cell lines SGC-7901 and MKN-28 were purchased from Genechem (Shanghai, China). HEK293T cells were purchased from ATCC (Manassas, VA, USA).

### YAP1 or SLC35B4 silencing in cell lines

The human YAP1-shRNA-1, human YAP1-shRNA-2, human SLC35B4-shRNA-1, and human SLC35B4-shRNA-2 were contracted into pLKO.1-TRC cloning vector (Addgene, Cambridge, MA, USA; 10878). The sequences of shRNAs were designed from sigma-Ardrich (St. Louis, MO, USA; Sigma-Ardrich TRC number: TRCN0000310615, TRCN0000300325, TRCN0000043982, TRCN0000370829), and the sequences were listed in supplementary materials Table 1. For generation of knockdown cell lines, lentivirus were generated using HEK293T cells. When the confluence of were 60~80%, cells were transfected with shRNA plasmid, packaging plasmid psPAX2 (Addgene, 12260), and envelope plasmid pMD2.G (Addgene, 12259) with the mass ratio of 4:3:1. The transfection of plasmids was using Lipofectamine Reagent 2000 (Invitrogen, Waltham, MA, USA) in antibiotic-free media. After 48 and 72 h later, the virus was harvested, and spun down at 1000 revolutions per minute (rpm), then purified using 0.45 micrometer (µm) syringe filter. For generation of YAP1/SLC35B4 stable knockdown cell lines, human GC cell lines SGC-7901 and MKN-28 were plated into six-well plates, and then incubated with lentivirus and cultured medium supplemented with eight microgram per milliliter (µg/ml) polybrene. After 24 h, cells were selected with 2 μg/ml puromycin for 3 days when there were no survival cells in the blank treatment groups.

### siRNA transfection

For siRNA knockdown experiments, cells were cultured into six-well plates overnight. Cells ware transfected with siRNAs using Lipofectamine Reagent 2000 (Invitrogen) in antibiotic-free media. Lipofectamine Reagent (1:1) and 200 micromole per liter (µm) of specific siRNAs were pre-mixed in the antibiotic-free media and incubated for 20 minutes (min) with room temperature. Cells were incubated for 6 h with Lipofectamine/siRNA mixture and cultured in fresh normal cell culture medium replaced. For mRNA analysis, cells were collected and total RNA was isolated after 48 h post transfection. siRNAs were designed using online BLOCK-iT RNAi Designer (https://rnaidesigner.thermofisher.com/rnaiexpress) and were synthetize by Genechem. siRNA sequences were listed in supplementary materials Table 2.

### Western blot assay

For detection, the protein levels of YAP1, phospho-Akt, Akt, phospho-Erk1/2, Erk1/2 and Actin, cells were collected after washing by phosphate-buffered saline (PBS) three times, and then disrupted by RIPA Lysis Buffer. The protein content were determined by BCA protein quantitative method. Proteins with different molecular weights were separated by sodium odecyl sulphate-polyacrylamide gel electrophoresis, and then transferred to nitrocellulose membrane. After blocking with 5% nonfat milk, the membranes were incubated with antibodies against YAP1 (Abcam, Cambridge, MA, USA; ab52771; 1:1 000), phospho-Akt (Cell Signaling Technology, Beverly, MA, USA; 4060 S; 1:1 000), Akt (Cell Signaling Technology; 9272; 1:1 000), phospho-Erk1/2 (Cell Signaling Technology; 9101 S; 1:1 000), Erk1/2 (Cell Signaling Technology; 9102 S; 1:1 000) and Actin (Sigma-Aldrich, St. Louis, MO,USA; A5316; 1:2 000) at 4 °C for 12 h. And incubated with the secondary antibodies. The proteins were detected with ECL chemiluminescent regents and visualized using Tanon 5500 (Tanon Science & Technology; Shanghai, China).

### Cell viability assay

Cells were plated in 96-wells plate with 1 × 10^3^ cells per well. Cell Counting Kit-8 assays were performed every day over the following 5 days. Each condition was replicated three times. Cells were incubated for 0.5~4 h in 100 µl culture medium with 10 µl Cell Counting Kit-8 (0.5 mg/ml) reagent at 37 °C. The absorbance was detected by Bio-RAD (Hercules, CA, USA) Microplate Reader at the wavelength of 450 nanometer (nm).

### Plate colony formation assay

Cells were cultured in six-well plate with 500–1000 cells per well and incubated for 2 weeks, and then fixed with 4% paraformaldehyde for 15 min and stained with 0.5% (w/v) crystal violet (Sigma-Aldrich) for 15 min. Colonies were photographed using Odyssey Scanner (LI-COR, Lincoln, NE, USA) and counted using ImageJ software.

### The soft agar colony formation assay

Cells were suspended in 0.3% low-melting agarose with normal cell culture medium, and plated onto a layer of 0.5% agrarose-containing medium in six-well plate (3000–5000 cells per well). Colonies were counted after 2–3 weeks, a phase-contrast microscopic pictures were taken for each samples using a digital camera coupled to a microscope.

### RNA isolation and quantitative real-time RT-PCR

Total RNA was isolated using TRIzol Reagent (Invitrogen). The first-strand of cDNA was synthesis by PrimeScript RT Master Mix (TaKaRa, Tokyo, Japan). Using the obtained cDNA as template, the quantitative real-time PCR was performed using SYBR-green PCR MasterMix (TaKaRa). Human β-actin gene was used as an internal control. PCR assays were performed three times, and the expression of genes was calculated using the comparative Ct method (ΔΔCt). Primers used for quantitative RT-qPCR were listed in supplementary materials Table 3.

### Microarray analysis

Expression profiling of control or YAP1-knockdown experimental samples in two cell lines (SGC-7901 and MKN-28) was performed using Affymetrix Genechip system. YAP1/SLC35B4 stable knockdown cell lines were harvested. Total RNA was extracted using TRIzol Reagent (Invitrogen) and purified with QIAGEN RNeasy Mini Kit (QIAGEN, Duesseldorf, Germany) and was quantified by the NanoDrop 2000 (Thermo Fisher Scientific). The RNA integrity was assessed using Agilent Bioanalyzer 2100 (Agilent Technologies, Santa Clara, CA, USA). The sample labeling, microarray hybridization and washing were performed based on the Affymetrix 3’IVT Expression microarray standard protocols. In brief, total RNA were used to synthesize cRNA and labeled with biotin. Standardized array processing procedures recommended by Affymetrix included hybridization, fluidics processing and scanning of the Affymetrix HG-U133 Plus 2.0 arrays. GeneSpring software (version 13.1; Agilent Technologies, Santa Clara, CA) was used to normalize the raw data (Affymetrix CEL files) by Robust Multichip Average algorithm. Differentially expressed genes were then identified through fold change. The threshold set for up- and downregulated genes was the fold change ≥2.0.

### Dual-luciferase report assay

The whole length of SLC35B4 promoter sequences and truncated sequences were cloned into the pGL3-Enhancer vector (Promega, Madison, WI, USA; E1771), which contains the firefly luciferase gene. Cells were seeded into 48-wells plate and transfected with 100 ng plasmids containing firefly luciferase reporters and 5 ng of plasmids expressing Renilla luciferase (pRL-TK vector, Promega, E2241) using Lipofectamine Reagent 2000 (Invitrogen) when the confluence of cells was 60~80%. After 48 h, the activity of luciferase was measured by the Dual-Luciferase Reporter Assay System (Promega). Firefly luciferase values were normalized by internal Renilla luciferase values.

### Tissue microarray analysis

The tissue microarrays were gained from Department of Pathology, Fourth Military Medical University. The microarray contained 60 samples of human gastric tumor. The tissue sections were performed immunohistochemistry assay with antibodies of YAP (Cell Signaling Technology, 14074; 1:100) and SLC35B4 (Sigma-Aldrich, HPA049779; 1:50). Immunohistochemistry photograph were scanned by Pannoramic (Santa Clara, CA, USA) MIDI and quantified with histochemistry score (H-Score) by Quant center. Correlation analysis of YAP and SLC35B4 was using GraphPad Prism (Version 6; La Jolla, CA, USA).

### Chromatin immunoprecipitation assay

Chromatin immunoprecipitation analysis of YAP was performed using the SimpleChIP Enzymatic Chromatin IP Kit (Cell Signaling Technology, 9003) as manufacturer’s protocol. SGC-7901 and MKN-28 cell lines were transfected with negative control RNA or siRNAs specific to YAP1. A total of 1.2 × 10^7^ cells were cross-linked with proteins in 1% formaldehyde for 10 min, and then lysed. The chromatin was harvested and digested with Micrococcal Nuclease into 150–900 bp DNA/protein fragments and purified. The purified chromatin from negative control or siYAP1-transfected cells were immunoprecipitated with antibody of YAP1 (Cell Signaling Technology, 14074) or IgG at 4 °C for 12 h. The proteins cross-linked with chromatin were degraded with Protease K and RNAase A, then the DNA was purified. The relative amount of interest chromatin regions in the immunoprecipitate was detected by qPCR. Primer sequences for qPCR amplification are listed in supplementary materials Table 4.

### Xenograft nude mice model

GC cell line SGC-7901 infected with YAP1-shRNA and control scramble shRNA were digested by trypsinase and then suspended to 1 × 10^7^ ml^−1^ cells after washing by PBS. BALB/c (nu/nu) nude mice ageing 4–6 weeks were subcutaneous injected with 200 µl cell suspension on the back. Tumor volumes were measured twice a week. The mice were killed after 4–5 weeks and the tumors were removed into polyformaldehyde solution for fixing tissues. The xenografts were taken to detect the volume and weight. The volume of tumor was calculated by 1/2 length × width^2^.

### Clinical data analysis

The data for mRNA levels of YAP1 and SLC35B4 in GC patients was downloaded from Gene Expression Omnibus (GEO, https://www.ncbi.nlm.nih.gov/geo/), and the threshold of fold change was set to 1.5. Normalized RNAseq data and follow-up data of GC were downloaded from The Cancer Genome Atlas (TCGA, http://gdac.broadinstitute.org/). The survival analysis of GC patients was done by Kaplan–Meier Plotter (http://kmplot.com/analysis/). Ranked expression values for YAP1 and SLC35B4 in stomach tumors from 450 patients from TCGA Stomach Cancer project provisional data set were downloaded for co-expression pattern analysis, and the linear regression analysis was performed by GraphPad Prism. *P* values were based on Spearman’s coefficient test.

### Statistical analysis

All experiments were repeated two to four times. The data from independent experiments were shown as mean ± SD. Survival curves were calculated using the Kaplan–Meier method. Statistical significance was determined by Student’s *t* test using GraphPad Prism, and differences were considered statistically significant when *P* value < 0.05 (*p < 0.05, **p < 0.01, ***p < 0.001).

## Supplementary information

Supplementary materials
