# Supplementary material for: A novel YAP1/SLC35B4 regulatory axis contributes to proliferation and progression of gastric carcinoma
Source: Cell Death Dis. 2019 Jun 7;10(6):452. doi: 10.1038/s41419-019-1674-2 (PMC6555804; doi:10.1038/s41419-019-1674-2)
Supplement: Supplementary file 1 — Supplementary materials [file 41419_2019_1674_MOESM1_ESM.doc]

**DNA and RNA sequences**

Table 1 Primers used for shRNA

| Target | Sigma-Ardrich TRC number | Sequences |
| --- | --- | --- |
| *YAP1*-1 | TRCN0000310615 | Forward primer: CCGGCAGGTGATACTATCAACCAAACTCGAGTTTGGTTGATAGTATCACCTGTTTTTG |
|  |  | Reverse primer: AATTCAAAAACAGGTGATACTATCAACCAAACTCGAGTTTGGTTGATAGTATCACCTG |
| *YAP1*-2 | TRCN0000300325 | Forward primer:  CCGGGCCACCAAGCTAGATAAAGAACTCGAGTTCTTTATCTAGCTTGGTGGCTTTTTG |
|  |  | Reverse primer:  AATTCAAAAAGCCACCAAGCTAGATAAAGAACTCGAGTTCTTTATCTAGCTTGGTGGC |
| *SLC35B4*-1 | TRCN0000043982 | Forward primer: CCGGCGGTTCTCTAATTGCCAACATCTCGAGATGTTGGCAATTAGAGAACCGTTTTTG |
|  |  | Reverse primer:  AATTCAAAAACGGTTCTCTAATTGCCAACATCTCGAGATGTTGGCAATTAGAGAACCG |
| *SLC35B4*-2 | TRCN0000370829 | Forward primer:  CCGGGAGACTCTCTACAAACGATTTCTCGAGAAATCGTTTGTAGAGAGTCTCTTTTTG |
|  |  | Reverse primer:  AATTCAAAAAGAGACTCTCTACAAACGATTTCTCGAGAAATCGTTTGTAGAGAGTCTC |

Table 2 siRNAs

| Target | Sequences |
| --- | --- |
| *YAP1* | CCACCAAGCUAGAUAAAGA |
|  | GGUCAGAGAUACUUCUUAA |
| *TEAD1* | GGCCGAUUUGUAUACCGAA |
|  | GGAUCAGACUGCAAAGGAU |
| *TEAD2* | CCUGGUGAAUUUCUUGCACAA |
|  | GGGAAAUCCAGUCCAAGUU |
| *TEAD3* | UACCUUGCUCUCAAUCUGGAG |
|  | GGAGACCCUGCUUGUCAUU |
| *TEAD4* | UUUCCUGCACACACGUCUCUU |
|  | CCGCCAAAUCUAUGACAAA |

Table 3 Primers used for qPCR

| Gene symbol | GenBank Accession | Forward primer | Reverse primer |
| --- | --- | --- | --- |
| *ALCAM* | [NM_001243281](http://www.ncbi.nlm.nih.gov/entrez/query.fcgi?cmd=Search&db=Nucleotide&term=NM_001243281) | ACTTGACGTACCTCAGAATCTCA | CATCGTCGTACTGCACACTTT |
| *ARHGAP18* | NM_033515 | ATCAAGAGGTGGTTGTTGTCAAA | ACAATGCTTTCCTGTGGATCTC |
| *CASC4* | [NM_177974](http://www.ncbi.nlm.nih.gov/entrez/query.fcgi?cmd=Search&db=Nucleotide&term=NM_177974) | CTTCCAGTCCTCTTCAGCGTT | CTGACTCTGTCCTTGGTAGCCT |
| *CDC42SE2* | [NM_020240](http://www.ncbi.nlm.nih.gov/entrez/query.fcgi?cmd=Search&db=Nucleotide&term=NM_020240) | TTCTGGTTGTGTTTCAACTGCT | GGCTCTCCAATCATACTTCTGTC |
| *CTGF* | [NM_001901](http://www.ncbi.nlm.nih.gov/entrez/query.fcgi?cmd=Search&db=Nucleotide&term=NM_001901) | GCACCAGCATGAAGACATACCG | CGTCAGGGCACTTGAACTCC |
| *DDAH1* | [NM_012137](http://www.ncbi.nlm.nih.gov/entrez/query.fcgi?cmd=Search&db=Nucleotide&term=NM_012137) | AACTCACTGTGCCTGATGACA | TTCCAGTTCAGACATGCTCACGG |
| *FAM208A* | [NM_001112736](http://www.ncbi.nlm.nih.gov/entrez/query.fcgi?cmd=Search&db=Nucleotide&term=NM_001112736) | GCATCTCTGACAGACACAGTC | CACAAGTAGGCTGCTCATAGTC |
| *MYCBP* | [NM_012333](http://www.ncbi.nlm.nih.gov/entrez/query.fcgi?cmd=Search&db=Nucleotide&term=NM_012333) | AATAGAGCTGCTTCGCCTAGA | GAGGTGGTTCATACTGAGCAAG |
| *ITGB3BP* | [NM_001206739](http://www.ncbi.nlm.nih.gov/entrez/query.fcgi?cmd=Search&db=Nucleotide&term=NM_001206739) | AATCTCCTGTGCATCACATTTCT | TTCATAGCTGTCAAGATGACGTG |
| *LAMP2* | [NM_001122606](http://www.ncbi.nlm.nih.gov/entrez/query.fcgi?cmd=Search&db=Nucleotide&term=NM_001122606) | AGATTCAGAAAATGCCACTTGCC | CTGATCATCCCCACAAATGCTT |
| *MPP7* | [NM_173496](http://www.ncbi.nlm.nih.gov/entrez/query.fcgi?cmd=Search&db=Nucleotide&term=NM_173496) | AGAACCACTGGGAGCTACCAT | CCCGTTGACTTCCCTAAGTTCAT |
| *MRPL17* | [NM_022061](http://www.ncbi.nlm.nih.gov/entrez/query.fcgi?cmd=Search&db=Nucleotide&term=NM_022061) | CGCGTGTGGACGAAATGAG | ACTCGATCACTGCCATCTTGG |
| *PYROXD1* | [NM_024854](http://www.ncbi.nlm.nih.gov/entrez/query.fcgi?cmd=Search&db=Nucleotide&term=NM_024854) | ACTGCTGGGAAAATACAATGCAC | AAGACAGCTCCCATCATTCGT |
| *SLC30A5* | [AF212235](http://www.ncbi.nlm.nih.gov/entrez/query.fcgi?cmd=Search&db=Nucleotide&term=AF212235) | ACCAAACACCAGTGGATCAAAA | CAGCAAAGTCCTTAGTGGTCC |
| *SLC35B4* | [NM_032826](http://www.ncbi.nlm.nih.gov/entrez/query.fcgi?cmd=Search&db=Nucleotide&term=NM_032826) | AAGCCACCAGCTATCCCAATA | GCATAGTTGTTCACCACGCTC |
| *TSPAN6* | [NM_003270](http://www.ncbi.nlm.nih.gov/entrez/query.fcgi?cmd=Search&db=Nucleotide&term=NM_003270) | ACTTGTTTCAAGAGCGTTCTGC | CAATGAGCACGAAGGGGACAT |
| *YAP1* | [NM_006106](http://www.ncbi.nlm.nih.gov/entrez/query.fcgi?cmd=Search&db=Nucleotide&term=NM_006106) | CTCACAGCAGAACCGTTTCCC | AGCCAAAACAGACTCCATGTCA |
| *TEAD1* | [NM_021961](http://www.ncbi.nlm.nih.gov/entrez/query.fcgi?cmd=Search&db=Nucleotide&term=NM_021961) | GCCTCCCAACATCCATAGCA | TCTGTCCACCAGCCGAGATT |
| *TEAD2* | [NM_001256660](http://www.ncbi.nlm.nih.gov/entrez/query.fcgi?cmd=Search&db=Nucleotide&term=NM_001256660) | TGCCTTCTTCCTGGTCAAGTTC | GGCTCTCATACTGGCTGCTCA |
| *TEAD3* | [NM_003214](http://www.ncbi.nlm.nih.gov/entrez/query.fcgi?cmd=Search&db=Nucleotide&term=NM_003214) | GCCGTCTTCTCCACTTCCTC | CCAGGGGCTCATAACTGCTG |
| *TEAD4* | [NM_201441](http://www.ncbi.nlm.nih.gov/entrez/query.fcgi?cmd=Search&db=Nucleotide&term=NM_201441) | GGGCAGACCTCAACACCAAC | TGTCCATTCTCATAGCGAGCA |

Table 4 Primers used for ChIP-qPCR

| Name | Forward primer | Reverse primer |
| --- | --- | --- |
| *CTGF* | GCCAATGAGCTGAATGGAGT | CAATCCGGTGTGAGTTGATG |
| *SLC35B4* | CAACGAGCAGTCACAGCTCT | AGCTCCGCCAGAGAGGACAG |
